# Unraveling Gut Microbiota Signatures Associated with PPARD and PARGC1A Genetic Polymorphisms in a Healthy Population

**DOI:** 10.3390/genes13020289

**Published:** 2022-02-01

**Authors:** María Bailén, Mariangela Tabone, Carlo Bressa, María Gregoria Montalvo Lominchar, Mar Larrosa, Rocío González-Soltero

**Affiliations:** MAS Microbiota Research Group, Universidad Europea de Madrid, Calle Tajo s/n, Villaviciosa de Odón, 28670 Madrid, Spain; maria.bailen@uam.es (M.B.); mariangela.tabone@universidadeuropea.es (M.T.); carlo.bressa@ufv.es (C.B.); goyi.montalvo@telefonica.net (M.G.M.L.); mlarrosa@ucm.es (M.L.)

**Keywords:** *PPARD*, *PPARGC1A*, microbiota, diabetes

## Abstract

Recent studies have revealed the importance of the gut microbiota in the regulation of metabolic phenotypes of highly prevalent metabolic diseases such as obesity, type II diabetes mellitus (T2DM) and cardiovascular disease. Peroxisome proliferator-activated receptors (PPARs) are a family of ligand-activated nuclear receptors that interact with PPAR-γ co-activator-1α (PPARGC1A) to regulate lipid and glucose metabolism. Genetic polymorphisms in *PPARD* (rs 2267668; A/G) and *PPARGC1A* (rs 8192678; G/A) are linked to T2DM. We studied the association between the single-nucleotide polymorphisms (SNPs) rs 2267668 and rs 8192678 and microbiota signatures and their relation to predicted metagenome functions, with the aim of determining possible microbial markers in a healthy population. Body composition, physical exercise and diet were characterized as potential confounders. Microbiota analysis of subjects with *PPARGC1A* (rs 8192678) and *PPARD* (rs 2267668) SNPs revealed certain taxa associated with the development of insulin resistance and T2DM. Kyoto encyclopedia of gene and genomes analysis of metabolic pathways predicted from metagenomes highlighted an overrepresentation of ABC sugar transporters for the *PPARGC1A* (rs 8192678) SNP. Our findings suggest an association between sugar metabolism and the *PPARGC1A* rs 8192678 (G/A) genotype and support the notion of specific microbiota signatures as factors related to the onset of T2DM.

## 1. Introduction

Recent studies attribute a central role to microbiota in the regulation of metabolic phenotypes related to highly prevalent metabolic diseases such as obesity, type 2 diabetes mellitus (T2DM) and many cardiovascular diseases [1,2,3]. This regulation is likely mediated by signaling molecules produced by the intestinal microbiota with potentially important effects at the level of the liver, including bile salts metabolism, cholesterol deposits and energy expenditure, and on insulin sensitivity in peripheral tissues [4]. The composition of the intestinal microbiota is influenced by several exogenous factors, such as diet, antibiotic intake and physical exercise, and also by endogenous factors, such as host genetics [5,6,7,8]. Indeed, host genetic variation can shape microbial diversity and the metabolites produced by these microorganisms [7,8,9], particularly in the case of specific genetic polymorphisms [9,10,11,12]. In this line, a pioneering genome-wide association study carried out on the Dutch LifeLines DEEP cohort identified different genetic loci that could directly influence the richness of the human microbiome [13]. Several of the 32 genetic markers identified in that study belong to the functional category of metabolic regulation.

Peroxisome proliferator-activated receptors (PPARs) are a family of ligand-activated nuclear receptors. Three PPAR protein subtypes of PPARs, α (PPARA), γ (PPARG) and δ (PPARD), have been identified with distinct roles in lipid and glucose metabolism. PPARα and γ, produced mainly in the liver and adipocytes, regulate fatty acid oxidation and adipogenesis, whereas *PPARD* is ubiquitously expressed, with higher levels reported in skeletal muscle and adipose tissue [14]. *PPARD* expression in skeletal muscle increases upon fasting [15] and during exercise [16], indicating a role in the adaptive response of skeletal muscle to the increased demand for fatty acid oxidation. In the liver, PPARD enhances glucose flux through the pentose-phosphate pathway and has insulin-sensitizing activity [17]. A genetic polymorphism in *PPARD* (rs 2267668; A/G intron variant) affects insulin sensitivity by modifying skeletal muscle glucose uptake [18] and also predicts the conversion from impaired glucose tolerance to T2DM [19]. The minor “G” allele has been associated with suppressed mitochondrial function in skeletal muscle [20]. In studies conducted in OLETF rats, a model of T2DM with obesity, the synthetic PPARD agonist GW0742 attenuated hepatic fat accumulation and improved insulin signaling [21], validating PPARD as a potential mediator of metabolic disease and a promising target for prevention and/or therapy.

PPAR-γ co-activator-1α (PPARGC1A) is a transcriptional coactivator that interacts with PPARs and functions as a master regulator of mitochondrial biogenesis and activity, including oxidative phosphorylation and reactive oxygen species detoxification [22]. PPARGC1A also plays a critical role in the maintenance of glucose and energy homeostasis and is likely involved in pathological disorders such as diabetes, neurodegeneration, obesity and cardiomyopathy [23]. Along this line, recent reports have suggested that the *PPARGC1A* Gly482Ser (rs 8192678; G/A coding sequence) missense polymorphism is associated with the onset of T2DM [24,25]. PPARD and PPARGC1A interact in the regulation of insulin action and modulate glucose and lipid metabolism in mitochondria during aerobic exercise [20].

The past decade has witnessed an explosion of interest in the gut microbiota as an important regulator of host metabolism as alterations in its composition, for example, by host genetics, are known to contribute to the development of obesity and insulin resistance [26]. Several studies support crosstalk between PPARs and the gut microbiota that influences the development of metabolic diseases, likely through the production of metabolites, such as short-chain fatty acids (SCFAs), which function as signaling molecules (reviewed in [27]). Some microbiota metabolites are also transported to the liver, adipose tissue, heart, blood vessels and other organs through systemic circulation. In these organs, the metabolites act as ligands of PPARs. The activation of PPARs modulates, between others, carbohydrate and fat metabolism [28].

In the present study, we searched for a relationship between single-nucleotide polymorphisms (SNPs) in *PPARD* and *PPARGC1A* and microbiota signatures and investigated the possible consequences at the phenotypic level, including glucose metabolism.

## 2. Materials and Methods

### 2.1. Ethics Approval and Consent to Participate

The study was performed in accordance with the Declaration of Helsinki, and the protocol was approved by the Research Ethics Committee of the Community of Madrid (CEIm-R; Ref: 47/560280.9/18). Written and informed consent was obtained from all participants.

### 2.2. Participant Characteristics

Seventy-six healthy participants (40 men and 36 women), aged 18–48 years, were included in the study. Exclusion criteria were any kind of pathology (during or six months prior to the study); previous gastrointestinal surgery; antibiotics intake during three months prior to the study; smoking; use of prebiotics, probiotics or nutritional complements; being vegetarian or vegan; and pregnancy or lactation. All participants were Caucasian.

### 2.3. Anthropometry and Body Composition

Height and weight were measured with a tallimeter (Asimed T2, Barcelona, Spain) and a balance scale (Ano Sayol SL, Barcelona, Spain), respectively and body mass index (BMI) was calculated as weight (kg)/height (m^2^). Body composition was evaluated on the day of stool sample collection by dual-energy X-ray absorptiometry (DEXA) (Hologic DEXA scan, Hologic Inc., Barcelona, Spain). Body composition measurements with DEXA were as follows: estimated visceral adipose tissue (VAT), body fat percentage (BFP), body fat mass (BFM), total lean mass, and fat and lean mass distribution in the trunk and extremities. The following indices were calculated using the obtained values: adiposity index (AI) = total fat/height^2^; muscular mass index (MMI) = total muscle mass/height^2^ and appendicular muscular mass index (AppMMI) = lean mass in arms + legs/height^2^.

### 2.4. Physical Activity

The levels of physical activity of the study population were recorded for one week (5 weekdays and 2 weekend days) with an ActiSleep V.3.4.2 accelerometer (Actigraph, Manufacturing Technology Inc., Shalimar, FL, USA). Acceleration, energy expenditure, the intensity of physical activity and body position were registered. The results were analyzed with Actilife software (Actilife6, Actigraph). Participants were instructed to wear the accelerometer on the right wrist all day except when they had a shower or performed pool activities. Data were considered valid when they contained a record of at least five valid days, including at least one weekend day and at least 10 h of activity. The time-sampling interval (epoch) was every minute during the 7 days. Physical activity was considered as light when the activity count was 100–1951 counts/minute, and moderate-to-vigorous when counts were 1952–5724 counts/minute according to the methodology described by Freedson et al. [29].

### 2.5. Dietary Habits

Dietary characterization was performed using a validated food frequency questionnaire (FFQ) with 93 food items, which was analyzed using DietSource software 3.0 (Novartis, Barcelona, Spain) to obtain the total energy macronutrients (fat, carbohydrates, fiber and proteins) and fiber intake.

### 2.6. Sample Collection

Participants were provided with the Fe-Col^®^ Fecal Sample Collection Kit (Alpha Laboratories, Hampshire, UK), an insulated bag and ice blocks to preserve the samples until they were delivered to the laboratory. Stool samples were stored at −80 °C until extraction.

### 2.7. Short-Chain Fatty Acids

Fecal SCFAs were extracted according to the protocol described by García-Villalba et al. [30]. For sample analyses, 1 µL of the supernatants was injected into an Agilent GC System 7820A chromatograph equipped with a DBWax 121-7037LT column and an Agilent Series MSD 5975 detector (Agilent Technologies, Inc., Santa Clara, CA, USA). Data acquisition was performed by selective ion monitoring. SCFAs were quantified using the peak area of their target ions against an eight-point external calibration curve (0.02 to 5.00 ppm) of reference standards (Sigma-Aldrich, St. Louis, MO, USA). 4-Methylvaleric acid was used as an internal standard.

### 2.8. DNA Extraction

Human and bacteria DNA were extracted from 100 mg of stool sample using the commercial E.Z.N.A.^®^ Stool DNA Kit (Omega Biotek, Norcross, GA, USA) and a bead-beating homogenizer (Bullet Blender Storm, Next Advance, New York, NY, USA). The concentration and purity of DNA were measured using the Quant-iT PicoGreen dsDNA Assay Kit (ThermoFisher Scientific, Waltham, MA, USA) and an FP-8300 spectrofluorimeter (Jasco, Tokyo, Japan). Bacterial DNA was used to analyze the microbiota, while human DNA was used for *PPARD* and *PPARGC1A* genotyping.

### 2.9. PPARD and PPARGC1A Genotyping

Allelic discrimination analysis was performed with predesigned Applied Biosystems TaqMan^®^ SNP Genotyping Assays: *PPARD* A/G (rs 2267668) (ID: C__15872729_10), *PPARGC1A* (rs 8192678) Gly482Ser (C/T) (ID: C___1643192_20) and the StepOnePlus Real-Time PCR system from ThermoFisher Scientific. The protocol included a denaturation stage at 95 °C for 10 min, 50 cycles of denaturation at 92 °C for 15 s, annealing/extension at 60 °C for 1 min and a final extension step of 30 s at 60 °C.

After genotyping, participants were classified according to the *PPARD* or *PPARGC1A* genotypes. For the *PPARD* A/G (rs 2267668) gene, those participants carrying the AA genotype were classified as PPARD-1 and AG as PPARD-2 for further analysis (participants with GG genotypes were not considered because of the low number.). In the case of the *PPARGC1A* (rs 8192678) Gly482Ser (C/T) gene, those participants with the CC genotype were classified as PPARGC1A-1 and the heterozygotes CT (TT participants were not considered as in PPARD for further analysis).

### 2.10. Sequencing and Bioinformatics

The hypervariable V3 and V4 regions were amplified using the primer pair 5′-TCGTCGGCAGCGTCAGATGTGTATAAGAGACAG-3′ and 5′-GTCTCGTGGGCTCGGAGATGTGTATAA GAGACAG-3′. The amplicon of 459 bp was visualized in a 0.8% agarose gel stained with ethidium bromide, and bands were cut and cleaned using the MinElute Gel Extraction Kit (Qiagen, Hilden, Germany). DNA amplicons were sequenced on a MiSeq Illumina platform (Illumina, San Diego, CA, USA). Sequence outputs were analyzed using the Quantitative Insights into Microbial Ecology (QIIME2) program, v2019.10 [31]. The 16s paired reads were imported in QIIME2 and processed with the DADA2 plugin [32], adjusting the maximum expected error threshold to 2.0 (both forward and reverse). The taxonomy assignments were performed with the classify-sklearn method [33] and an in-house customized classifier based on the SILVA reference database [34,35]. To build the customized reference database, sequences, according to our primers (forward primer sequence: CCTACGGGNGGCWGCAG, reverse primer sequence: GACTACHVGGGTATCTAATCC), were extracted from the SILVA 132 database clustered at 99% identity. The classifier was trained using our tailored reference reads and SILVA 7-levels for reference taxonomy, including the species probability (weights) likely to be observed for human stool (downloaded from https://github.com/BenKaehler/readytowear, accessed on 10 September 2020) [36,37]. Diversity analyses were performed through QIIME 2′s q2-diversity plugin. β-diversity was evaluated by calculating the Bray–Curtis, Jaccard, unweighted and weighted Unifrac distance metrics. To study α-diversity, observed operational taxonomic units (OTUs), evenness and Shannon and Faith’s Phylogenetic Diversity indices were calculated. The Kyoto Encyclopedia of Genes and Genomes (KEGG) ortholog abundances predictions were obtained with the Phylogenetic Investigation of Communities by Reconstruction of Unobserved States (PICRUSt2) software [38] using default “max parsimony” method for hidden-state prediction and a Nearest Sequenced Taxon Index (NSTI) value of 2.0.

### 2.11. Statistical Analysis

Statistical analysis was carried out using QIIME2 v2019.10, SPSS software v26.0 (SPSS, Chicago, IL, USA) and the R statistical package v4.1.1. Variable normal distribution was assessed using the Shapiro–Wilk test; non-parametric tests were performed when normal distribution was not assumed. Comparisons of variables were performed with t-tests or the Mann–Whitney test. Linear discriminant analysis coupled with effect size (LEfSe v1.0) was performed to identify bacterial associated pathways differentially represented between groups with default settings. Significance was set at *p* < 0.05. To verify whether the allele frequencies were in Hardy–Weinberg equilibrium for the *PPARD* genotypes, we used the Chi-square (χ^2^) test. Differential abundance analysis was carried out using the edgeR algorithm [39]. The *p*-value was corrected for multiple testing using the Benjamini–Hochberg false discovery rate (FDR) procedure, and the results were considered as significant for FDR < 0.001.

## 3. Results

### 3.1. Subjects, Genotypes and Allelic Frequencies

Seventy-six people were recruited for the study (40 men and 36 women). Participants were genotyped for *PPARD* (rs 2267668) and *PPARGC1A* (rs 8192678), and the proportions of genotypes and allelic frequencies were calculated (Table 1). Participants with the genotype *PPARD* (rs 2267668) G/G were not included in the gut microbiota study due to the low number (three participants).

To verify that the genotypes were in accordance with other populations previously reported to be in Hardy–Weinberg equilibrium, we applied the χ^2^ test to compare experimental and expected data. No significant differences were found for *PPARD* rs 2267668 (Gly482Ser) carriers, indicating that our population was in equilibrium for this SNP. Conversely, significant differences were found for *PPARGC1A* (rs 8192678), which is likely because we used a healthy population rather than a disease-related group such as a T2DM population where the mutant allele is overrepresented [40].

### 3.2. Body Composition, Physical Activity and Dietary Habits

We next examined for effects of the polymorphisms on body composition, physical activity and dietary habits. Age and body composition parameters were analyzed according to sex (Table S1) and genotype (Table 2).

Body composition parameters did not differ between groups when participants were classified according to the *PPARD* or *PPARGC1A* genotypes except for the MMI, which was significantly greater in subjects with *PPARGC1A* genotype 2 (PPAGC1A-2) than in those with genotype 1 (PPARGC1A-1). No significant differences were found in energy expenditure and intensity of physical activity during weekdays or weekends according to the *PPARD* (genotypes PPARD-1 or PPARD-2) or *PPARGC1A* genotype.

Foods and food groups were recorded from the FFQ and analyzed to obtain energy, macronutrients and fiber intake. No significant differences between the *PPARD* and *PPARGC1A* genotypes were observed for any of the studied macronutrients (carbohydrates, protein, fat, protein/carbohydrate, protein/fat ratio), fiber and total energy intake (Table S2).

### 3.3. Short-Chain Fatty Acids

No within-group differences were found for fecal levels of SCFAs (acetic acid, propionic acid, butyric acid, isobutyric acid, valeric acid and isovaleric acid) according to the polymorphisms studied (Table S3).

### 3.4. Fecal Microbiota

The average number of reads per sample was 100,818. No significant differences in α-diversity parameters were observed between PPARGC1A-1 and PPARGC1A-2: observed OTUs (*p* = 0.810), Shannon index (*p* = 0.345), Pielou evenness (*p* = 0.114) or Faith’s Phylogenetic Diversity index (*p* = 0.969) (Figures S1–S4); or between PPARD-1 and PPARD-2: observed OTUs (*p* = 0.954), Shannon index (*p* = 0.810), Pielou evenness (*p* = 0.715) or Faith’s Phylogenetic Diversity index (*p* = 0.854) (Figures S5–S8). Likewise, no significant differences were observed for β-diversity for *PPARGC1A* or *PPARD* in relation to genotypes (Figures S9 and S10): PPARGC1A; Bray–Curtis (*p* = 0.313), Jaccard (*p* = 0.690), unweighted Unifrac (*p* = 0.790) and weighted Unifrac (*p* = 0.222) distance metrics. PPARD; Bray–Curtis (*p* = 0.837), Jaccard (*p* = 0.733), unweighted Unifrac (*p* = 0.65) and weighted Unifrac (*p* = 0.693) distance metrics.

### 3.5. Differential Abundance Analysis

**Figure 1:**
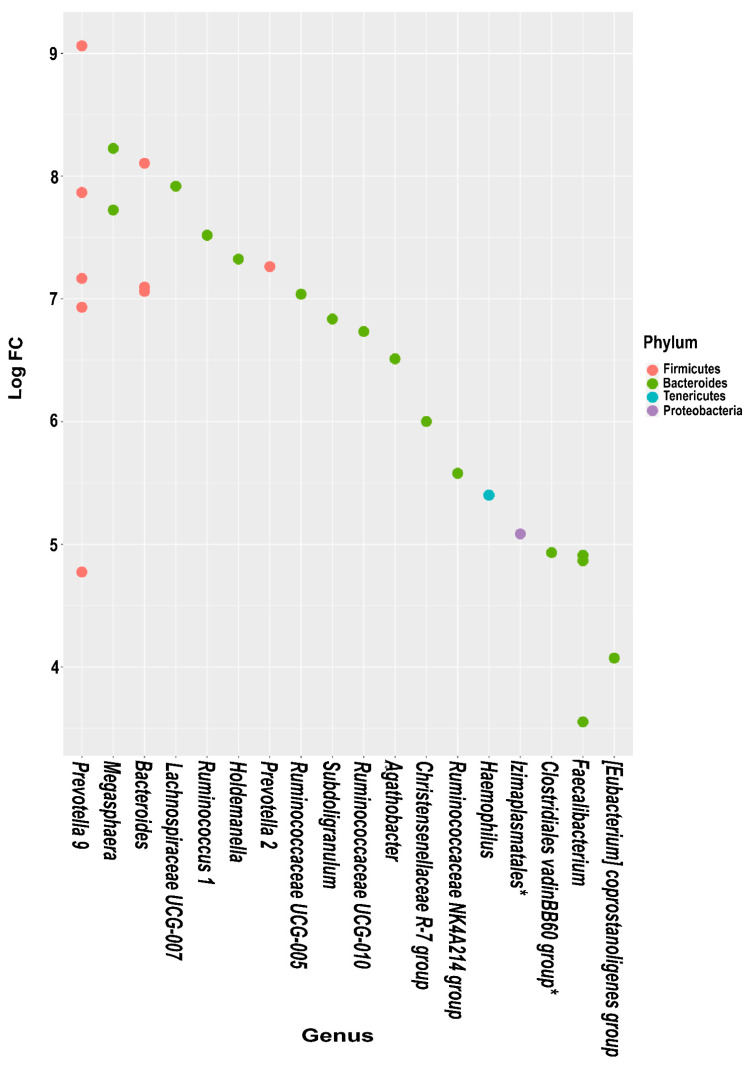
Log2-fold-change of the relative abundance of individual operational taxonomic units of PPARGC1A-1 compared PPARGC1A-2 genotypes at the phylum and genus level. * Identification at order or family level.

**Figure 2:**
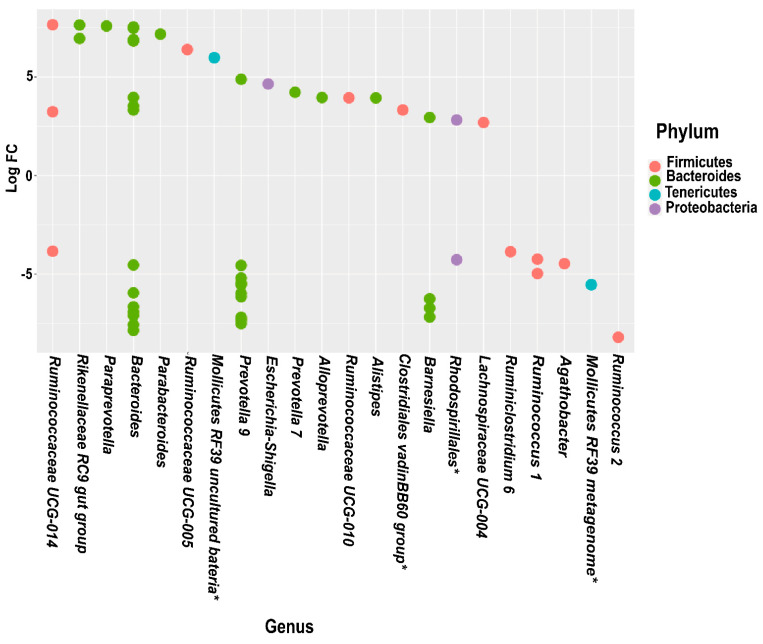
Log2-fold-change of the relative abundance of individual operational taxonomic units of PPARD-1 compared to PPARD-2 genotypes at the phylum and genus level. * Identification at order or family level.

Differential abundance analysis revealed variations at several taxonomic levels for the *PPARGC1A* and *PPARD* polymorphisms (Figure 1 and Figure 2). Of note, *Bacteriodetes* and *Firmicutes* were the phyla that showed the most significant variations between PPARGC1A-2 and PPARGC1A-1; more specifically, *Prevotella 9*, *Megasphaera*, *Bacteroides* and *Lachnospiraceae UCG-007* genera. Analysis of *PPARD* polymorphisms indicated that *Bacteriodetes* was the phylum with more differences between the *PPARD* genotypes (Figure 2). The microbiota of participants with PPARD-1 showed an overrepresentation of *Ruminiclostridium 6*, *Ruminococcus* 1, *Agathobacter*, *Prevotella* 9 and *Barnesiella*, whereas the microbiota of peers with PPARD-2 was enriched in three genera of the *Ruminicoccaceae* family, *Rikenellaceae* RC9 gut group, *Paraprevotella*, *Parabacteroides*, *Escherichia*, *Alloprevotella* and *Alistipes*.

### 3.6. Predicted Functional Metagenome by PICRUSt

**Figure 3:**
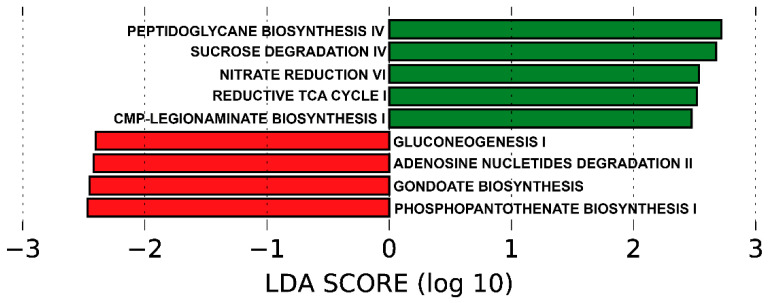
Predicted functional composition of metagenomes based on 16SrRNA gene sequencing data. LEfSe based on the PICRUSt2 dataset revealed differentially enriched metabolic pathways associated with *PPARGC1A* genotypes 1 and 2 (PPARGC1A-1 (red); PPARGC1A-2 (green)). LDA: linear discriminant analysis.

The functionality of the different metagenomes, grouped by genotypes for *PPARGC1A* (PPARGC1A-1 and PPARGC1A-2) and *PPARD* (PPARD-1 and PPARD-2), was predicted using PICRUSt, which uses the 16S rRNA reads to predict functional pathway abundance. No differences between metabolic pathways were identified based on *PPARD* genotypes. However, for *PPARGC1A*, PICRUSt analysis predicted several pathways associated with the PPARGC1A-1 or PPARGC1A-2 genotypes (Figure 3).

**Figure 4:**
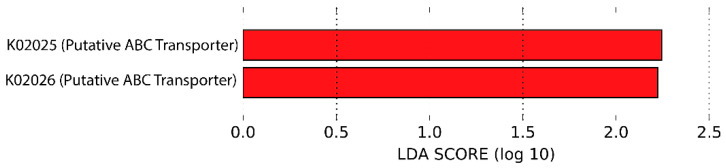
KEGG metabolic pathways predicted from metagenomes based on 16SrRNA gene sequenced data (red: genotype 2: PPARG1A-2). LDA: linear discriminant analysis.

The functional pathways overrepresented in genotype PPARGC1A-1 versus genotype PPARGC1A-2 were as follows: gluconeogenesis I, adenosine nucleotides degradation II, gondoate biosynthesis and phosphopantothenate biosynthesis I. By contrast, the functional pathways more present in genotype PPARGC1A-2 and less represented in genotype PPARGC1A-1 were as follows: peptidoglycan biosynthesis IV, sucrose degradation IV, nitrate reduction VI, reductive TCA cycle I and CMP-legionaminate biosynthesis I. Analysis of the KEGG metabolic pathways predicted from metagenomes revealed an overrepresentation of ABC sugar transporters in participants with the PPARG1A-2 genotype when compared with those presenting the PPARG1A-1 genotype (Figure 4).

## 4. Discussion

The establishment of the gut microbiota is a multifactorial process influenced by host genetics, diet and physical exercise, among other factors, and is now considered to have an important role in the onset of several metabolic diseases [5,6,7,8]. Indeed, a genome-wide association study in humans reported a large number of genetic polymorphisms associated with increased risk of obesity and diabetes, with each gene, individually, seeming to have a small summative effect [41].

*PPARGC1A* is a transcriptional coactivator of the PPAR family that regulates insulin sensitivity and influences the onset of T2DM [42]. Poor insulin sensitivity or insulin resistance is a predictor of T2DM and is observed in hypertension, dyslipidemia and cardiovascular diseases [43,44,45]. Insulin resistance in skeletal muscle has been attributed to several pathological conditions such as mitochondrial dysfunction [46], impaired glycogen synthesis [47] and the accumulation of diacylglycerol, with subsequent impairment of insulin signaling [48]. The *PPARGC1A* SNP rs 8192678 (in exon 8, G1444A/Gly482Ser; C/T) is the most important polymorphism identified to date in *PPARGC1A* [49]. The Gly482Ser (T) allele has been associated with poorer mitochondrial function in skeletal muscle [20], with an increased risk of T2DM [24], and the presence of the minor T allele seems to be overrepresented in studies focused on T2DM susceptibility [40]. However, there is controversy over this claim, as some studies have reported an increased risk [50,51], and others have failed to demonstrate a significant effect or have even observed a decreased risk of T2DM in the heterozygotes (CT) and homozygotes (TT) of this polymorphism [49,52,53,54,55]. One of the reasons for the disparity of results could be the disparity of populations studied; some of them were performed in populations with a higher risk of developing T2DM, where the prevalence of the minor allele is probably overrepresented when compared with the general population. Further studies in well-established samples from healthy populations are needed to have a more accurate estimation of the risk due this genetic factor in a particular context.

Gut microbiota may have a role in this interplay between genetics and the onset of metabolic disorders, probably through microbial metabolites [56]. We found that the microbiota of participants with the PPARGC1A-2 genotype was enriched in *Prevotella* 9, *Megasphaera*, *Bacteroides* and *Lachnospiraceae* UCG-007 genera, which have previously been associated with insulin resistance and diabetes. For example, *Megasphaera* and *Prevotella* 9 have been linked to prediabetes in an Indian population [57], in whom *Megasphaera* exhibited significant positive correlations with fasting plasma glucose and glycated hemoglobin levels and weak negative correlations with high-density lipoprotein, suggesting an influence on susceptibility to diabetes for carriers of the minor Ser(T) allele. Additionally, advanced stage type 1 diabetic (T1DM) rats had a higher relative abundance of *Prevotella* 9 and *Bacteroides* [58]. Of note, the *Lachnospiraceae* family has been reported to actively impair glucose metabolism, leading to inflammation and promoting the onset of type 1 diabetes [59], and other metagenomics studies have shown an association between *Lachnospiraceae* and T2DM in both humans and in mouse models [60,61,62].

Microbial metabolites from the gut microbiota have important roles in maintaining host metabolic homeostasis. PICRUSt analysis can infer associations between gut microbiota and host metabolic changes and functional capabilities. Focusing on the relevant functional categories highlighted by PiCRUSt, we found that the genotype PPARGC1A-2 was positively associated with sucrose phosphorylase enzymes, which function in the metabolism of sucrose [63]. Sucrose metabolism could be a factor implicated in the multifactorial etiology of T2DM and a possible source of dysbiosis [64]. Carriers of the PPARG1A-2 genotype also showed an overrepresentation of the oxoglutarate synthase pathway. 2-Oxoglutarate is a fundamental metabolic intermediate that is key for regulating the tricarboxylic acid cycle (TCA). In addition, 2-oxoglutarate acts as the major carbon skeleton for nitrogen-assimilatory reactions, indicating that intracellular levels of 2-oxoglutarate fluctuate according to nitrogen and carbon availability [65]. Bacteria in the gut produce various metabolites, such as amino acids, SCFAs, bile acids, indole propionic acids, trimethylamine and nitrogen oxide, which mediate host metabolism and are essential to maintain an intact intestinal barrier. The microbiota of patients with T2DM is characterized by a reduced number of butyrate-producing bacteria [66,67]. Nevertheless, we failed to find significant differences in SCFAs between genotypes for the two genes studied.

Based on the KEGG pathways analysis, carriers of PPARGC1A-2 showed an overrepresentation of ABC transporters. An association between membrane transporters (ABC transporters) and obesity has recently been reported [68]. ABC transporters are linked to the transport of glucose and lipids, and this is currently being exploited to treat T2DM, for example, gliflozin, a sodium-glucose co-transporter 2 (SGLT2) inhibitor. Several transporter families, including SLC2A (GLUT, ABCT) and SLC5A (SGLT) transporters, are either linked to glucose or with the homeostasis of cholesterol [69]. ABC transporters and butyrate and acetate levels are significantly enriched in the microbiota of genetically determined obesity (ob/ob mice), likely because of a specialization of the gut microbiota to increase the capacity to harvest energy from the diet [70]. However, we failed to find differences in the production of butyrate or acetate between the different genotypes.

Beyond the genetic factors, intrinsic factors, such as dietary habits or physical activity levels, could influence the presence of different microbiota patterns. Participants in our study did not show differences in potential confounders such as diet, physical exercise or body composition in relation to genotypes of the studied genes with the exception of MMI related to PPARGC1A polymorphisms. This difference might be explained by the small number of women carrying the Gly482Ser allele in our sample.

Genetic polymorphism in *PPARD* (rs2267668; A/G: PPARD-2) has also been described to affect insulin sensitivity and the conversion from impaired glucose tolerance to T2DM [18]. Several studies have reported gut microbiota dysbiosis as a factor in the rapid progression of insulin resistance in T2DM. The gut microbiota of participants with the PPARD-2 genotype showed a decrease in *Prevotella* 9 and *Barnesiella*. A lower abundance of *Prevotella* has been reported in T2DM [71,72], and *Barnesiella* has been found to decrease in obesity [73]. However, results obtained with *Prevotella* 9 are controversial as it has been found to increase [57] or decrease [71,72] in T2DM in different studies. Beneficial and pathogenic effects of members of *Prevotella* could be related to distinct roles of different species ([57,72,74]. Likewise, *Alistipes*, *Parabacteroides* and *Escherichia*, enriched in PPARD-2 participants, were also increased in T2DM, correlating with less butyrate-producing bacteria and more opportunistic pathogens (*Clostridium, Escherichia*) [60,75,76]. Nonetheless, *Parabacteroides* conversely decreases in T2DM patients in a study conducted in China [67].

A limitation of the present study was the small sample size because of the unequal distribution of genotypes in the population, which ruled out the possibility of studying the two genes and associated polymorphisms together. Our findings reveal an association between different microbiota features, sugar metabolism (ABC transporters) and the *PPARGC1A* Gly482Ser genotype and some microbiota features associated with the PPARD-2 genotype. The sum of all these genetics and microbiota-related factors likely interact and contribute to the onset of diabetes or pre-diabetes-like phenotypes; however, further studies are needed to provide insight into these interactions. Future studies should examine the relationship between these microbiota taxa and host physiology factors as glucose and insulin levels, or the Homeostatic Model Assessment for Insulin Resistance (HOMA index), as potential early predictive markers of T2DM. More studies focusing on healthy populations are needed to establish a stratification risk, which would be important for the prevention of non-transmissible diseases in a particular genetic context. For this purpose, the synergistic factors of host genetics and microbiota could be used as biomarkers to better predict the onset of the disease in patients with prediabetes. Our studies constitute a first step in establishing the role of gut microbiota in the complex scenario of multifactorial and highly prevalent non-transmissible diseases.

## Figures and Tables

**Table 1:** Genotypes and allelic proportions.

	*PPARD* (rs 2267668)				*PPARGC1A* (rs 8192678)			
	Genotype Frequency	Expected Frequency *	Allelic Frequencies		Genotype Frequency	Expected Frequency **	Allelic Frequencies	
AA	0.62	0.67	Allele A	0.79	CC 0.934	0.445	Allele C	0.967
AG	0.34	0.29	Allele G	0.21	CT 0.066	0.444	Allele T	0.033
GG	0.039	0.035			TT 0	0.111		

* (European origin; source: SNPedia). ** (European origin; source: [40]).

**Table 2:** Age, sex and body composition parameters of participants according to their genotype.

	PPARD-1	PPARD-2	*p*	PPARGC1A-1	PPARGC1A-2	*p*
Sex (*n*/%)	23/50 M 23/50 W	15/55.6 M 12/44.4 W	0.796	36/51.4 M 34/48.6 W	4/66.7 M 2/33.3 W	0.677 *
Age (years)	33.73 ± 7.40	33.73 ± 8.06	1.00	33.26 ± 7.87	36.83 ± 2.04	0.27
Body mass (kg)	69.25 ± 13.05	70.27 ± 12.20	0.75	69.22 ± 12.93	73.75 ± 8.11	0.40
BMI (kg/m^2^)	24.21 ± 3.61	23.77 ± 3.12	0.61	23.94 ± 3.53	25.40 ± 1.42	0.32
BFP (%)	26.07 ± 7.48	27.82 ± 8.56	0.39	27.21 ± 7.90	23.22 ± 6.26	0.28
BFM (kg)	17.36 ± 6.59	18.95 ± 6.02	0.33	18.15 ± 6.47	17.03 ± 4.90	0.71
VAT (g)	332.98 ± 192.11	356.20 ± 181.26	0.63	343.53 ± 179.66	368.80 ± 257.95	0.77
AI (kg/m^2^)	6.06 ± 2.12	6.4 ± 2.32	0.45	6.32 ± 2.20	5.76 ± 1.69	0.58
MMI (kg/m^2^)	16.05 ± 2.21	15.77 ± 2.57	0.63	15.77 ± 2.36	17.98 ± 1.41	0.04 **
AppMMI (kg/m^2^)	7.17 ± 1.29	6.99 ± 1.42	0.60	7.02 ± 1.35	8.08 ± 0.74	0.09

BMI: body mass index; BFP: body fat percentage; BFM: body fat mass; VAT: estimated visceral fat; AI: adiposity index; MMI: muscular mass index; AppMMI: appendicular muscular mass index. M: men; W: women. Values are mean ± standard deviation. PPARD-1: PPARD genotype 1. PPARD-2: PPARD genotype 2. PPARGC1A-1: PGC1-α genotype 1. PPARGC1A-2: PGC1-α genotype 2. * Fisher’s exact test. ** *p* < 0.05.

## Data Availability

The datasets presented in this study can be found in online repositories. The names of the repository/repositories and accession number(s) can be found below: NCBI BioProject; Accession No. PRJNA799142.

## Supplementary Materials

The following supporting information can be downloaded at: https://www.mdpi.com/article/10.3390/genes13020289/s1. Table S1: Age and body composition parameters of participants according to gender; Table S2: Total energy, macronutrients and fiber dietary intake; Table S3: Short-chain fatty acids in fecal samples form PPARGC1A and polymorphisms; Figure S1: Observed features and PPARGC1A polymorphisms; Figure S2: Shannon index and PPARGC1A polymorphisms; Figure S3: Pielou evenness and PPARGC1A polymorphisms; Figure S4: Faith diversity and PPARGC1A polymorphisms; Figure S5: Observed features and PPARD polymorphisms; Figure S6: Shannon index and PPARD polymorphisms; Figure S7: Pielou evenness and PPARD polymorphisms; Figure S8: Faith diversity and PPARD polymorphisms; Figure S9: Principal Coordinate Analysis (PcoA) plots of unweighted (A) and weighted (B) Unifrac distance metrics Bray–Curtis (C) and Jaccard for PPARGC1A polymorphisms; Figure S10: Principal Coordinates Analysis (PcoA) plots of unweighted (A) and weighted (B) Unifrac distance metrics Bray–Curtis (C) and Jaccard for PPARD polymorphisms.

## References

[1] Schoeler M., Caesar R. (2019). Dietary lipids, gut microbiota and lipid metabolism. Rev. Endocr. Metab. Disord..

[2] Bäckhed F., Ding H., Wang T., Hooper L.V., Koh G.Y., Nagy A., Semenkovich C.F., Gordon J.I. (2004). The gut microbiota as an environmental factor that regulates fat storage. Proc. Natl. Acad. Sci. USA.

[3] Witkowski M., Weeks T.L., Hazen S.L. (2020). Gut Microbiota and Cardiovascular Disease. Circ. Res..

[4] Sonnenburg J.L., Bäckhed F. (2016). Diet–microbiota interactions as moderators of human metabolism. Nature.

[5] Cerdá B., Pérez M., Pérez-Santiago J.D., Tornero-Aguilera J.F., González-Soltero R., Larrosa M. (2016). Gut Microbiota Modification: Another Piece in the Puzzle of the Benefits of Physical Exercise in Health?. Front. Physiol..

[6] Bressa C., Bailén-Andrino M., Pérez-Santiago J., González-Soltero R., Pérez M., Montalvo-Lominchar M.G., Maté-Muñoz J.L., Domínguez R., Moreno D., Larrosa M. (2017). Differences in gut microbiota profile between women with active lifestyle and sedentary women. PLoS ONE.

[7] Spor A., Koren O., Ley R. (2011). Unravelling the effects of the environment and host genotype on the gut microbiome. Nat. Rev. Microbio..

[8] Blekhman R., Goodrich J.K., Huang K., Sun Q., Bukowski R., Bell J.T., Spector T.D., Keinan A., Ley R.E., Gevers D. (2015). Host genetic variation impacts microbiome composition across human body sites. Genome Biol..

[9] Khachatryan Z.A., Ktsoyan Z.A., Manukyan G.P., Kelly D., Ghazaryan K.A., Aminov R.I. (2008). Predominant Role of Host Genetics in Controlling the Composition of Gut Microbiota. PLoS ONE.

[10] Li E., Hamm C.M., Gulati A.S., Sartor R.B., Chen H., Wu X., Zhang T., Rohlf F.J., Zhu W., Gu C. (2012). Inflammatory Bowel Diseases Phenotype, *C. difficile* and NOD2 Genotype Are Associated with Shifts in Human Ileum Associated Microbial Composition. PLoS ONE.

[11] Tong M., McHardy I., Ruegger P., Goudarzi M., Kashyap P.C., Haritunians T., Li X., Graeber T.G., Schwager E., Huttenhower C. (2014). Reprograming of gut microbiome energy metabolism by the FUT2 Crohn’s disease risk polymorphism. ISME J..

[12] Knights D., Silverberg M.S., Weersma R.K., Gevers D., Dijkstra G., Huang H., Tyler A.D., Van Sommeren S., Imhann F., Stempak J.M. (2014). Complex host genetics influence the microbiome in inflammatory bowel disease. Genome Med..

[13] Bonder M.J., Kurilshikov A., Tigchelaar E.F., Mujagic Z., Imhann F., Vila A.V., Deelen P., Vatanen T., Schirmer M., Smeekens S.P. (2016). The effect of host genetics on the gut microbiome. Nat. Genet..

[14] Skogsberg J., Kannisto K., Roshani L., Gagné E., Hamsten A., Larsson C., Ehrenborg E. (2000). Characterization of the human peroxisome proliferator activated receptor delta gene and its expression. Int. J. Mol. Med..

[15] Holst D., Luquet S., Nogueira V., Kristiansen K., Leverve X., Grimaldi P.A. (2003). Nutritional regulation and role of peroxisome proliferator-activated receptor δ in fatty acid catabolism in skeletal muscle. Biochim. Biophys. Acta (BBA)-Mol. Cell Biol. Lipids.

[16] Russell A.P., Hesselink M.K.C., Lo S.K., Schrauwen P. (2005). Regulation of metabolic transcriptional co-activators and transcription factors with acute exercise. FASEB J..

[17] Lee C.-H., Olson P., Hevener A., Mehl I., Chong L.-W., Olefsky J.M., Gonzalez F.J., Ham J., Kang H., Peters J.M. (2006). PPAR regulates glucose metabolism and insulin sensitivity. Proc. Natl. Acad. Sci. USA.

[18] Vänttinen M., Nuutila P., Kuulasmaa T., Pihlajamäki J., Hällsten K., Virtanen K.A., Lautamäki R., Peltoniemi P., Takala T., Viljanen A.P.M. (2005). Single Nucleotide Polymorphisms in the Peroxisome Proliferator–Activated Receptor δ Gene Are Associated with Skeletal Muscle Glucose Uptake. Diabetes.

[19] Andrulionyte L., Peltola P., Chiasson J.-L., Laakso M., STOP-NIDDM Study Group (2006). Single Nucleotide Polymorphisms of PPARD in Combination with the Gly482Ser Substitution of PGC-1A and the Pro12Ala Substitution of PPARG2 Predict the Conversion from Impaired Glucose Tolerance to Type 2 Diabetes: The STOP-NIDDM Trial. Diabetes.

[20] Stefan N., Thamer C., Staiger H., Machicao F., Machann J., Schick F., Venter C., Niess A., Laakso M., Fritsche A. (2007). Genetic Variations inPPARDandPPARGC1ADetermine Mitochondrial Function and Change in Aerobic Physical Fitness and Insulin Sensitivity during Lifestyle Intervention. J. Clin. Endocrinol. Metab..

[21] Lee M.Y., Choi R., Kim H.M., Cho E.J., Kim B.H., Choi Y.S., Naowaboot J., Lee E.Y., Yang Y.C., Shin J.Y. (2012). Peroxisome proliferator-activated receptor δ agonist attenuates hepatic steatosis by anti-inflammatory mechanism. Exp. Mol. Med..

[22] Rius-Pérez S., Torres-Cuevas I., Millán I., Ortega Á.L., Pérez S. (2020). PGC-1α, Inflammation, and Oxidative Stress: An Integrative View in Metabolism. Oxid. Med. Cell. Longev..

[23] Lin J., Handschin C., Spiegelman B.M. (2005). Metabolic control through the PGC-1 family of transcription coactivators. Cell Metab..

[24] Ek J., Andersen G., Urhammer S.A., Gæde P., Drivsholm T., Borch-Johnsen K., Hansen T., Pedersen O. (2001). Mutation analysis of peroxisome proliferator-activated receptor-γ coactivator-1 (PGC-1) and relationships of identified amino acid polymorphisms to Type II diabetes mellitus. Diabetologia.

[25] Hara K., Tobe K., Okada T., Kadowaki H., Akanuma Y., Ito C., Kimura S. (2002). A genetic variation in the PGC-1 gene could confer insulin resistance and susceptibility to Type II diabetes. Diabetologia.

[26] Ussar S., Griffin N.W., Bezy O., Fujisaka S., Vienberg S., Softic S., Deng L., Bry L., Gordon J.I., Kahn C.R. (2015). Interactions between Gut Microbiota, Host Genetics and Diet Modulate the Predisposition to Obesity and Metabolic Syndrome. Cell Metab..

[27] Oh H.Y.P., Visvalingam V., Wahli W. (2019). The PPAR–microbiota–metabolic organ trilogy to fine-tune physiology. FASEB J..

[28] Hasan A.U., Rahman A., Kobori H. (2019). Interactions between Host PPARs and Gut Microbiota in Health and Disease. Int. J. Mol. Sci..

[29] Freedson P.S., Melanson E., Sirard J. (1998). Calibration of the Computer Science and Applications, Inc. accelerometer. Med. Sci. Sports Exerc..

[30] García-Villalba R., Bastida J.A.G., Conesa M.T.G., Tomas-Barberan F., Espín J.C., Larrosa M. (2012). Alternative method for gas chromatography-mass spectrometry analysis of short-chain fatty acids in faecal samples. J. Sep. Sci..

[31] Bolyen E., Rideout J.R., Dillon M.R., Bokulich N.A., Abnet C.C., Al-Ghalith G.A., Alexander H., Alm E.J., Arumugam M., Asnicar F. (2019). Reproducible, interactive, scalable and extensible microbiome data science using QIIME 2. Nat. Biotechnol..

[32] Callahan B.J., Mcmurdie P.J., Rosen M.J., Han A.W., Johnson A.J.A., Holmes S.P. (2016). DADA_2_: High-resolution sample inference from Illumina amplicon data. Nat. Methods.

[33] Pedregosa FABIANPEDREGOSA F., Michel V., Grisel OLIVIERGRISEL O., Blondel M., Prettenhofer P., Weiss R., Vanderplas J., Cournapeau D., Pedregosa F., Varoquaux G. (2011). Scikit-Learn: Machine Learning in Python Gaël Varoquaux Bertrand Thirion Vincent Dubourg Alexandre Passos PEDREGOSA, VAROQUAUX, GRAMFORT ET AL. Matthieu Perrot. J. Mach. Learn. Res..

[34] Quast C., Pruesse E., Yilmaz P., Gerken J., Schweer T., Yarza P., Peplies J., Glöckner F.O. (2013). The SILVA ribosomal RNA gene database project: Improved data processing and web-based tools. Nucleic Acids Res..

[35] Yilmaz P., Parfrey L.W., Yarza P., Gerken J., Pruesse E., Quast C., Schweer T., Peplies J., Ludwig W., Glöckner F.O. (2013). The SILVA and “All-species Living Tree Project (LTP)” taxonomic frameworks. Nucleic Acids Res..

[36] Bokulich N.A., Kaehler B.D., Rideout J.R., Dillon M., Bolyen E., Knight R., Huttley G.A., Gregory Caporaso J. (2018). Optimizing taxonomic classification of marker-gene amplicon sequences with QIIME 2’s q2-feature-classifier plugin. Microbiome.

[37] Kaehler B.D., Bokulich N., Mcdonald D., Knight R., Caporaso J., Gregory C.J., Huttley G.A. (2019). Species-Level Microbial Sequence Classification Is Improved by Source-Environment Information. bioRxiv.

[38] Douglas G.M., Maffei V.J., Zaneveld J.R., Yurgel S.N., Brown J.R., Taylor C.M., Huttenhower C., Langille M.G.I. (2020). PICRUSt2 for prediction of metagenome functions. Nat. Biotechnol..

[39] Anders S., McCarthy D.J., Chen Y., Okoniewski M., Smyth G.K., Huber W., Robinson M.D. (2013). Count-based differential expression analysis of RNA sequencing data using R and Bioconductor. Nat. Protoc..

[40] Prior S.L., Clark A.R., Jones D.A., Bain S.C., Hurel S.J., Humphries S.E., Stephens J.W. (2012). Association of the PGC-1? rs 8192678 Variant with Microalbuminuria in Subjects with Type 2 Diabetes Mellitus. Dis. Markers.

[41] Karaderi T., Drong A.W., Lindgren C.M. (2015). Insights into the Genetic Susceptibility to Type 2 Diabetes from Genome-Wide Association Studies of Obesity-Related Traits. Curr. Diabetes Rep..

[42] Lai C.-Q., Tucker K.L., Parnell L.D., Adiconis X., García-Bailo B., Griffith J., Meydani M., Ordovás J.M. (2007). PPARGC1A Variation Associated with DNA Damage, Diabetes, and Cardiovascular Diseases: The Boston Puerto Rican Health Study. Diabetes.

[43] Lillioja S., Mott D.M., Spraul M., Ferraro R., Foley J.E., Ravussin E., Knowler W.C., Bennett P.H., Bogardus C. (1993). Insulin Resistance and Insulin Secretory Dysfunction as Precursors of Non-Insulin-Dependent Diabetes Mellitus: Prospective Studies of Pima Indians. N. Engl. J. Med..

[44] Martin B.C., Warram J.H., Krolewski A.S., Soeldner J.S., Kahn C.R., Bergman R.N. (1992). Role of glucose and insulin resistance in development of type 2 diabetes mellitus: Results of a 25-year follow-up study. Lancet.

[45] Bloomgarden Z.T. (1998). Insulin resistance: Current concepts. Clin. Ther..

[46] Kim J.A., Wei Y., Sowers J.R. (2008). Role of Mitochondrial Dysfunction in Insulin Resistance. Circ. Res..

[47] Sesti G. (2006). Pathophysiology of insulin resistance. Best Pract. Res. Clin. Endocrinol. Metab..

[48] Petersen K.F., Shulman G.I. (2006). Etiology of Insulin Resistance. Am. J. Med..

[49] Csép K., Szigeti E., Vitai M., Koranyi L. (2017). The Ppargc1A-Gly482Ser Polymorphism (RS8192678) and the Metabolic Syndrome in a Central Romanian Population. Acta Endocrinol..

[50] Zhu S., Liu Y., Wang X., Wu X., Zhu X., Li J., Ma J., Gu H.F., Liu Y. (2009). Evaluation of the association between the PPARGC1A genetic polymorphisms and type 2 diabetes in Han Chinese population. Diabetes Res. Clin. Pract..

[51] Bhat A., Koul A., Rai E., Sharma S., Dhar M.K., Bamezai R.N.K. (2007). PGC-1α Thr394Thr and Gly482Ser variants are significantly associated with T2DM in two North Indian populations: A replicate case-control study. Qual. Life Res..

[52] Esterbauer H., Oberkofler H., Linnemayr V., Iglseder B., Hedegger M., Wolfsgruber P., Paulweber B., Fastner G., Krempler F., Patsch W. (2002). Peroxisome Proliferator-Activated Receptor-γ Coactivator-1 Gene Locus: Associations with Obesity Indices in Middle-Aged Women. Diabetes.

[53] Vohl M.-C., Houde A., Lebel S., Hould F.-S., Marceau P. (2005). Effects of the peroxisome proliferator-activated receptor-γ co-activator-1 Gly482Ser variant on features of the metabolic syndrome. Mol. Genet. Metab..

[54] Lacquemant C., Chikri M., Boutin P., Samson C., Froguel P. (2002). No association between the G482S polymorphism of the proliferator-activated receptor-gamma coactivator-1 (PGC-1) gene and Type II diabetes in French Caucasians. Diabetologia.

[55] Stumvoll M., Fritsche A., T’Hart L.M., Machann J., Thamer C., Tschritter O., Van Haeften T.W., Jacob S., Dekker J.M., Maassen J.A. (2004). The Gly482Ser Variant in the Peroxisome Proliferator-Activated Receptor γ Coactivator-1 is not Associated with Diabetes-Related Traits in Non-Diabetic German and Dutch Populations. Exp. Clin. Endocrinol. Diabetes.

[56] Franson J., Grose J., Larson K., Bridgewater L. (2021). Gut Microbiota Regulates the Interaction between Diet and Genetics to Influence Glucose Tolerance. Medicines.

[57] Pinna N.K., Anjana R.M., Saxena S., Dutta A., Gnanaprakash V., Rameshkumar G., Aswath S., Raghavan S., Rani C.S.S., Radha V. (2021). Trans-ethnic gut microbial signatures of prediabetic subjects from India and Denmark. Genome Med..

[58] Gao H., Jiang Q., Ji H., Ning J., Li C., Zheng H. (2019). Type 1 diabetes induces cognitive dysfunction in rats associated with alterations of the gut microbiome and metabolomes in serum and hippocampus. Biochim. Biophys. Acta (BBA)-Mol. Basis Dis..

[59] Kostic A., Gevers D., Siljander H., Vatanen T., Hyötyläinen T., Hämäläinen A.-M., Peet A., Tillmann V., Pöhö P., Mattila I. (2015). The Dynamics of the Human Infant Gut Microbiome in Development and in Progression toward Type 1 Diabetes. Cell Host Microbe.

[60] Qin J., Li Y., Cai Z., Li S., Zhu J., Zhang F., Liang S., Zhang W., Guan Y., Shen D. (2012). A metagenome-wide association study of gut microbiota in type 2 diabetes. Nature.

[61] Kameyama K., Itoh K. (2014). Intestinal Colonization by a Lachnospiraceae Bacterium Contributes to the Development of Diabetes in Obese Mice. Microbes Environ..

[62] Vacca M., Celano G., Calabrese F.M., Portincasa P., Gobbetti M., De Angelis M. (2020). The Controversial Role of Human Gut Lachnospiraceae. Microorganisms.

[63] Reid S.J., Abratt V.R. (2005). Sucrose utilisation in bacteria: Genetic organisation and regulation. Appl. Microbiol. Biotechnol..

[64] Hashimoto Y., Hamaguchi M., Kaji A., Sakai R., Osaka T., Inoue R., Kashiwagi S., Mizushima K., Uchiyama K., Takagi T. (2020). Intake of sucrose affects gut dysbiosis in patients with type 2 diabetes. J. Diabetes Investig..

[65] Huergo L., Dixon R. (2015). The Emergence of 2-Oxoglutarate as a Master Regulator Metabolite. Microbiol. Mol. Biol. Rev..

[66] Arora A., Behl T., Sehgal A., Singh S., Sharma N., Bhatia S., Sobarzo-Sanchez E., Bungau S. (2021). Unravelling the involvement of gut microbiota in type 2 diabetes mellitus. Life Sci..

[67] Li Q., Chang Y., Zhang K., Chen H., Tao S., Zhang Z. (2020). Implication of the gut microbiome composition of type 2 diabetic patients from northern China. Sci. Rep..

[68] Hou Y.-P., He Q.-Q., Ouyang H.-M., Peng H.-S., Wang Q., Li J., Lv X.-F., Zheng Y.-N., Li S.-C., Liu H.-L. (2017). Human Gut Microbiota Associated with Obesity in Chinese Children and Adolescents. BioMed Res. Int..

[69] Behl T., Sehgal A., Grover M., Singh S., Sharma N., Bhatia S., Al-Harrasi A., Aleya L., Bungau S. (2021). Uncurtaining the pivotal role of ABC transporters in diabetes mellitus. Environ. Sci. Pollut. Res..

[70] Turnbaugh P.J., Ley R.E., Mahowald M.A., Magrini V., Mardis E.R., Gordon J.I. (2006). An obesity-associated gut microbiome with increased capacity for energy harvest. Nature.

[71] Candela M., Biagi E., Soverini M., Consolandi C., Quercia S., Severgnini M., Peano C., Turroni S., Rampelli S., Pozzilli P. (2016). Modulation of gut microbiota dysbioses in type 2 diabetic patients by macrobiotic Ma-Pi 2 diet. Br. J. Nutr..

[72] Kovatcheva-Datchary P., Nilsson A., Akrami R., Lee Y.S., De Vadder F., Arora T., Hallen A., Martens E., Björck I., Bäckhed F. (2015). Dietary Fiber-Induced Improvement in Glucose Metabolism Is Associated with Increased Abundance of Prevotella. Cell Metab..

[73] Thingholm L.B., Rühlemann M.C., Koch M., Fuqua B., Laucke G., Boehm R., Bang C., Franzosa E.A., Hübenthal M., Rahnavard G. (2019). Obese Individuals with and without Type 2 Diabetes Show Different Gut Microbial Functional Capacity and Composition. Cell Host Microbe.

[74] Pedersen H.K., Gudmundsdottir V., Nielsen H.B., Hyotylainen T., Nielsen T., Jensen B.A.H., Forslund K., Hildebrand F., Prifti E., Falony G. (2016). Human gut microbes impact host serum metabolome and insulin sensitivity. Nature.

[75] Chen J., Zhao J., Cao Y., Zhang G., Chen Y., Zhong J., Huang W., Zeng J., Wu P. (2019). Relationship between alterations of urinary microbiota and cultured negative lower urinary tract symptoms in female type 2 diabetes patients. BMC Urol..

[76] Tao S., Li L., Li L., Liu Y., Ren Q., Shi M., Liu J., Jiang J., Ma H., Huang Z. (2019). Understanding the gut–kidney axis among biopsy-proven diabetic nephropathy, type 2 diabetes mellitus and healthy controls: An analysis of the gut microbiota composition. Geol. Rundsch..

