# Supplementary material for: Unraveling Gut Microbiota Signatures Associated with PPARD and PARGC1A Genetic Polymorphisms in a Healthy Population
Source: Genes (Basel). 2022 Feb 1;13(2):289. doi: 10.3390/genes13020289 (PMC8871880; doi:10.3390/genes13020289)

SUPPLEMENTARY MATERIAL

Table S1. Age and body composition parameters of participants according to their gender

	MEN			WOMEN			p
Age	34.10	±	7.84	32.94	±	7.43	0.58
Body mass	76.92	±	10.79	61.20	±	8.82	0.00*
BMI	24.80	±	2.87	23.21	±	3.83	0.01*
BFP	22.40	±	5.92	32.79	±	5.85	0.00*
BFM	17.08	±	6.32	19.36	±	6.25	0.07
VAT	386.82	±	196.96	291.47	±	152.47	0.05*
AI	5.519	±	2.00	7.26	±	1.97	0.00*
MMI	17.62	±	1.40	13.74	±	1.33	0.00*
AppMMI	8.086	±	0.74	5.82	±	0.69	0.00*

Table S2. Total energy, macronutrients and fiber dietary intake

	**PPARD-1**	**PPARD-2**	**p**	**PPARGC1 A-1**	**PPARGC1 A-2**	**p**
Energy (kcal/day)	2058.99±566.16	2301.93±843.30	0.15	2135.01±676.87	2294.63±669.20	0.58
Carbohydrates (% of energy)	45.77±6.05	46.31±6.18	0.72	45.64±6.24	46.17±4.92	0.84
Protein (% of energy)	17.16±2.89	17.96±3.22	0.29	17.45±2.90	16.67±4.03	0.54
Fa (% of energy)	37.00±6.25	35.88±6.34	0.48	36.88±6.45	37.33±5.92	0.87
Protein/carbohidrates	0.38±0.09	0.40±0.11	0.54	0.39±0.10	0.37±0.09	0.51
Protein/fat	0.48±0.13	0.52±0.16	0.25	0.49±0.14	0.47±0.18	0.64
Fiber (g/day)	23.76±11.39	23.52±8.79	0.93	23.32±10.20	25.15±12.74	0.68

Values are mean ± standard deviation PPARD-2: PPARD genotype 2. PPARGC1A-1: PPARGC1A genotype 1. PPARGC1A-2: PPARGC1A genotype 2 SFA: saturated fatty acids; MUFA: monounsaturated fatty acids; PUFA: polyunsaturated fatty acids.

Table S3. Short-chain fatty acids

**SCFA (μg/g)**	**PPARD1**	**PPARD2**	**P**	**PPGC1A-1**	**PPGC1A-2**	**P**
Acetic acid	158.25 ± 9.58	164.49 ± 19.55	0.377	159.75 ± 9.56	152.54 ± 29.98	0.98

Propionic acid	79.94 ± 5.73	87.53 ± 9.59	0.707	81.70 ± 4.96	82.81 ± 24.63	0.87
Isobutyric acid	15.51 ± 1.80	15.36 ± 1.43	0.398	15.55 ± 1.29	12.37 ± 2.39	0.66
Butyric acid	91.67 ± 7.75	117.83 ± 17.16	0.25	101.12 ± 8.29	81.21 ± 22.42	0.60
Isovaleric acid	28.01 ± 2.99	30.59 ± 3.44	0.234	29.14 ± 2.34	23.97 ± 4.77	0.76
Valeric acid	22.02 ± 1.94	24.08 ± 2.24	0.224	22.07 ± 1.45	26.75 ± 6.09	0.36
Total SFCA	395.3870 ± 24.15	439.89 ± 44.43	0.736	409.32 ± 22.57	379.66 ± 86.00	0.87

Values are mean ± standard deviation PPARD-2: PPARD genotype 2. PPARGC1A-1: PPARGC1A genotype 1. PPARGC1A-2: PPARGC1A genotype 2

Figure S1. Observed features and *PPARGC1A* polymorphisms

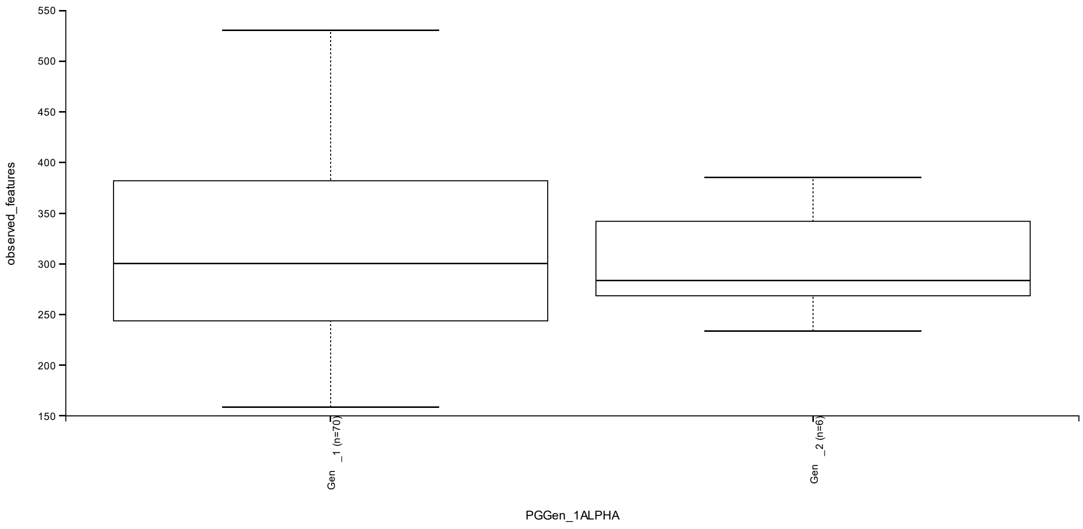

Figure S2. Shannon index and *PPARGC1A* polymorphisms

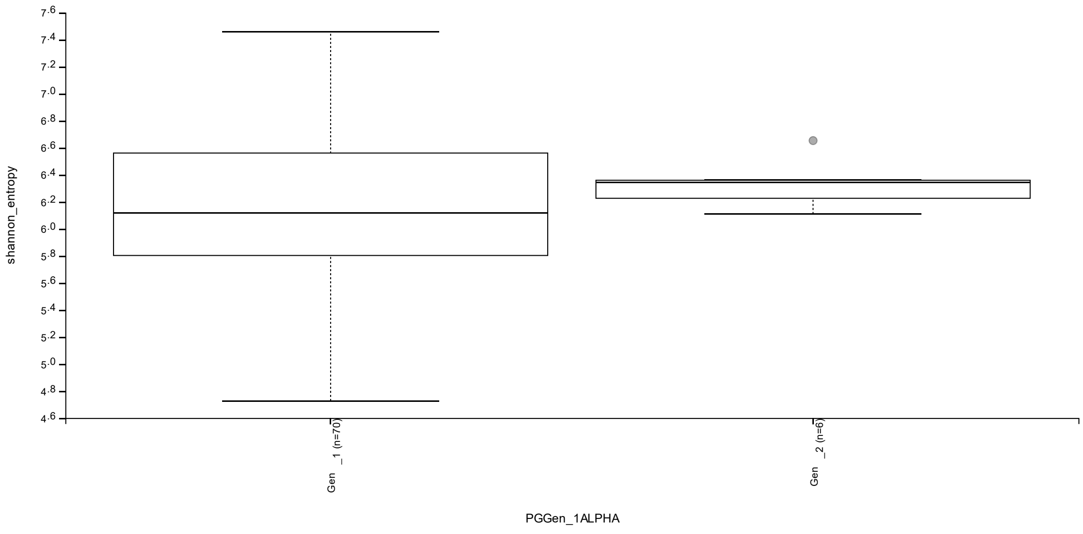

Figure S3. Pielou evenness and *PPARGC1A* polymorphisms

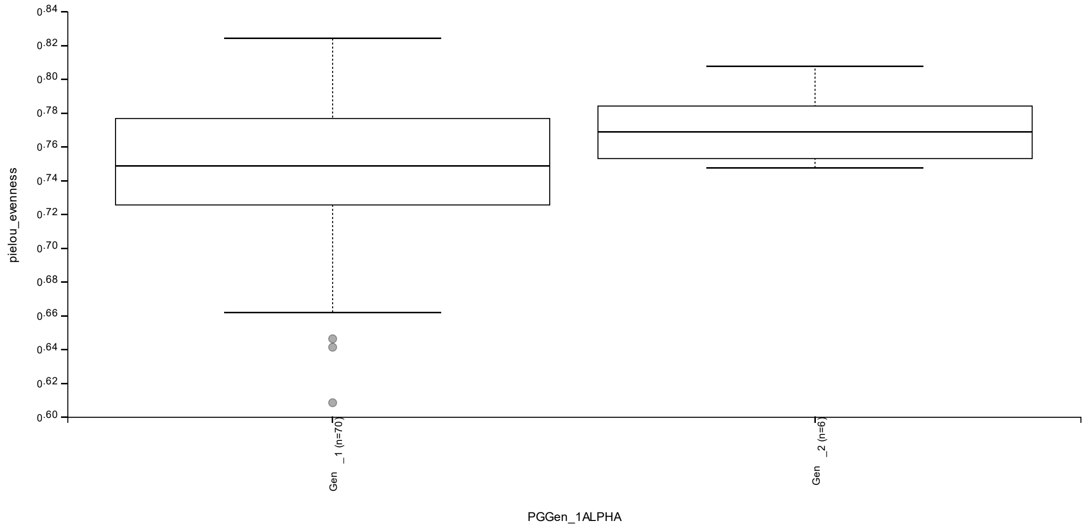

Figure S4. Faith diversity and *PPARGC1A* polymorphisms

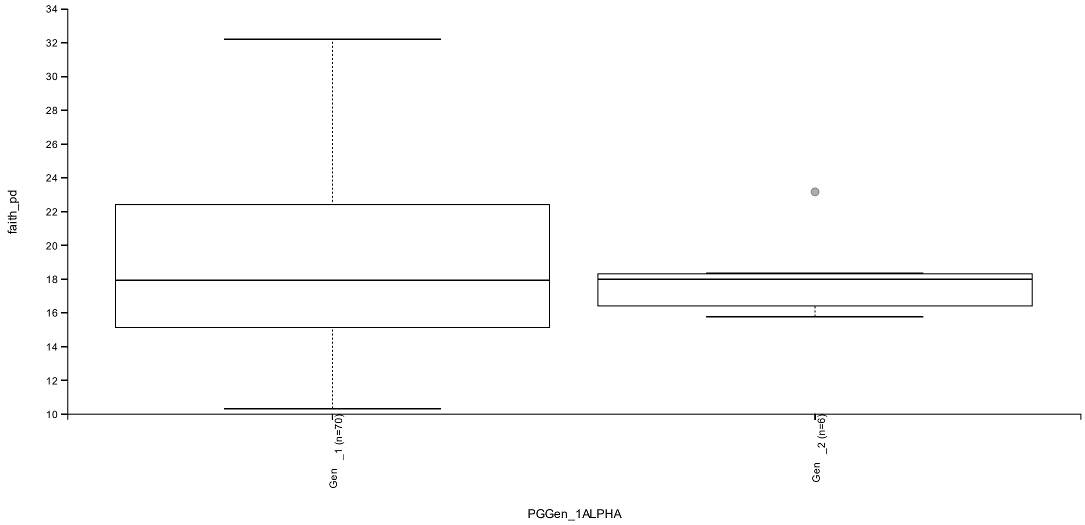

Figure S5. Observed features and *PPARD* polymorphisms

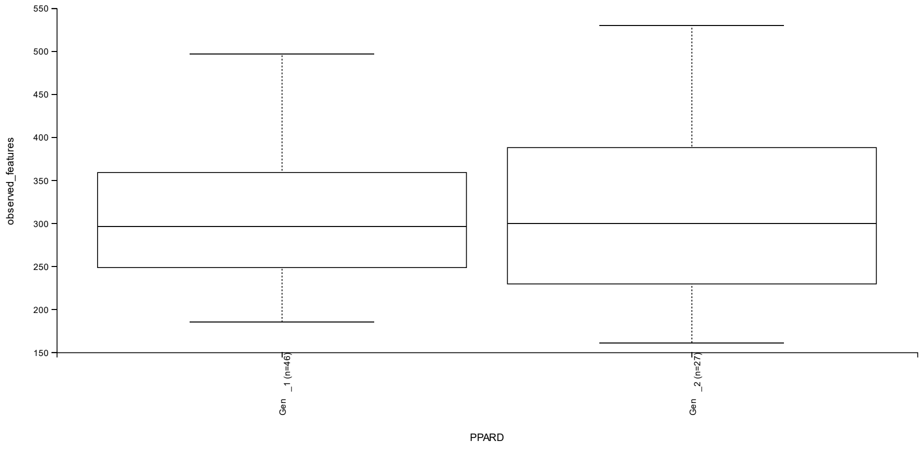

Figure S6. Shannon index and *PPARD* polymorphisms

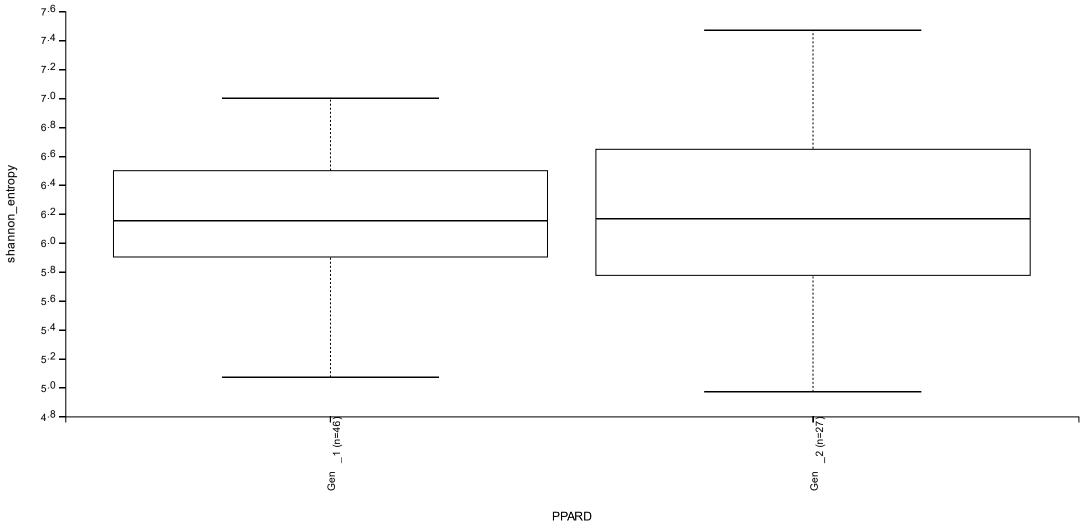

Figure S7. Pielou evenness and PPARD polymorphisms

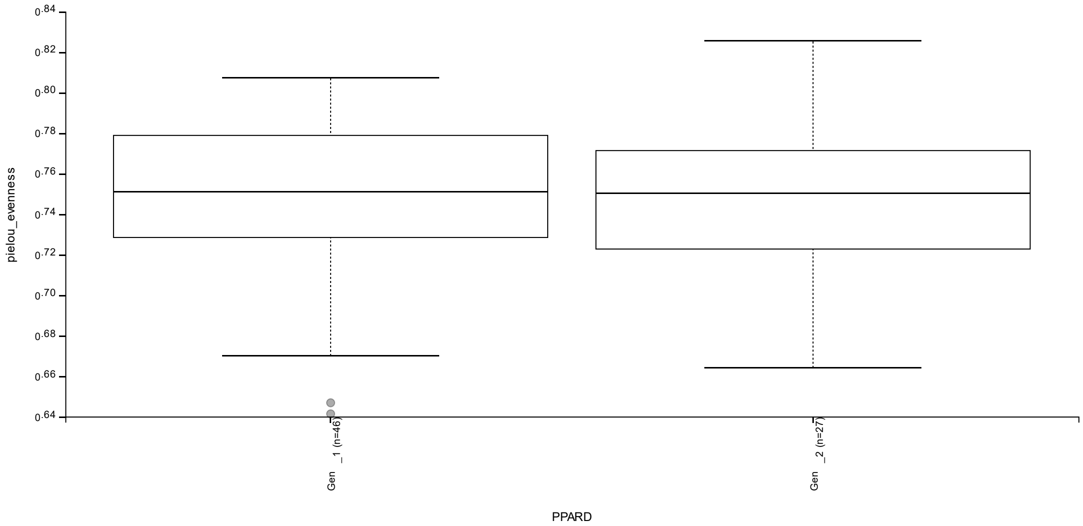

Figure S8. Faith diversity and *PPARD* polymorphisms

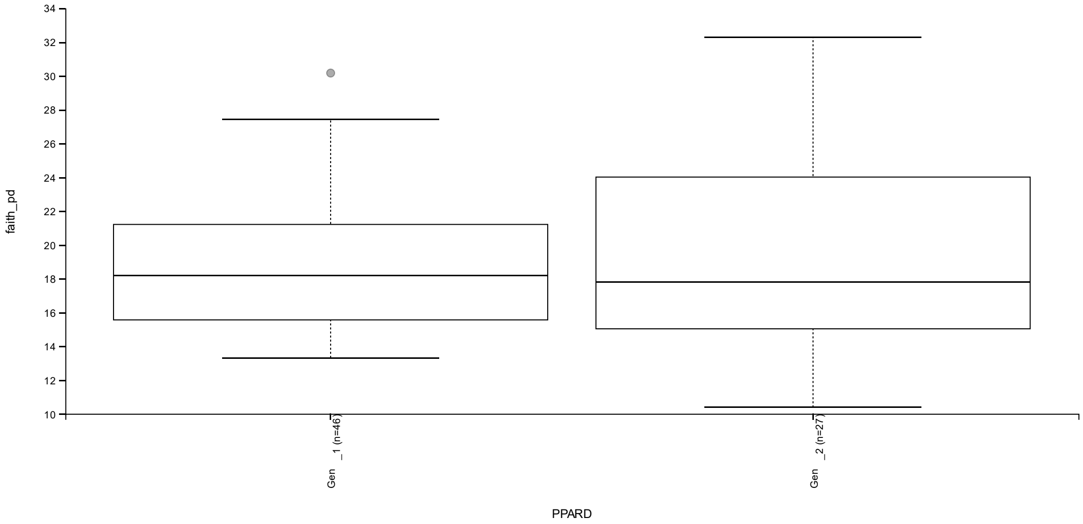

Figure S9. Principal coordinates analysis plots of unweighted (A) and weighted (B) Unifrac distance metrics Bray-Curtis (C) and Jaccard (D) for *PPARGC1A* polymorphisms. Red: genotype 1; Blue: genotype 2

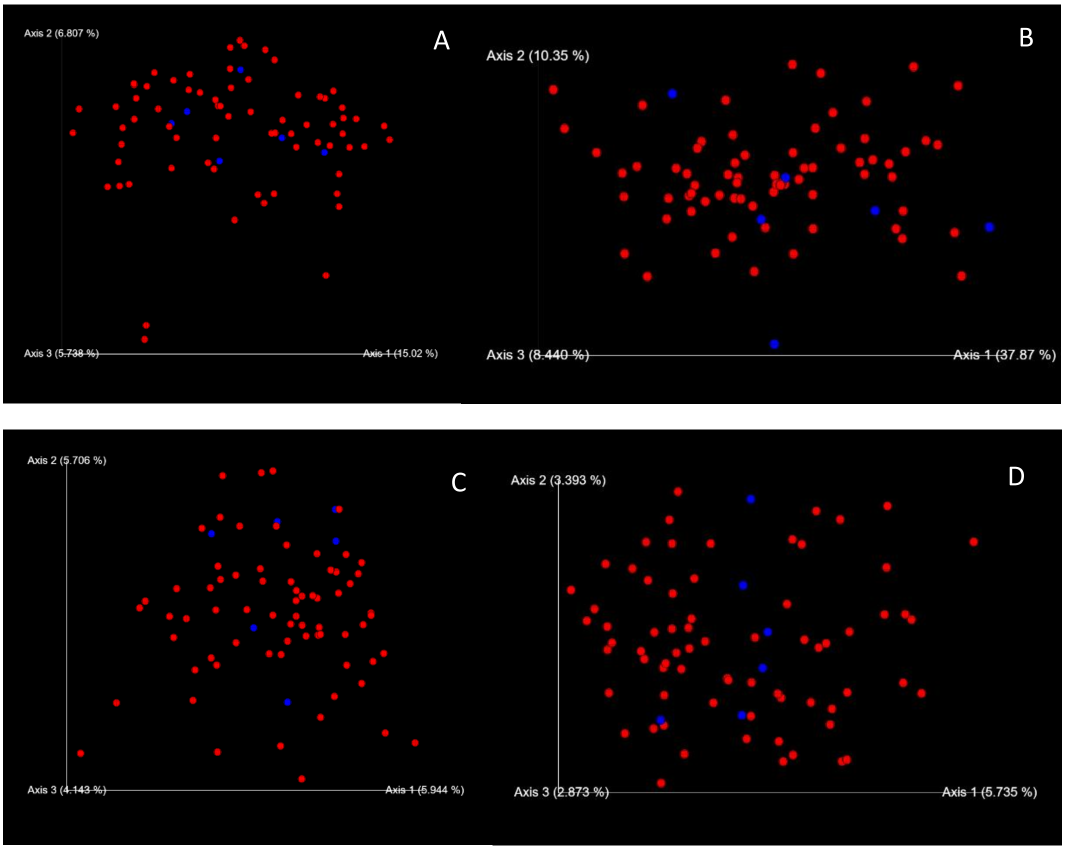

Figure S10. Principal coordinates analysis plots of unweighted (A) and weighted (B) Unifrac distance metrics Bray-Curtis (C) and Jaccard (D) for *PPARD* polymorphisms. Red: genotype 1; Blue: genotype 2

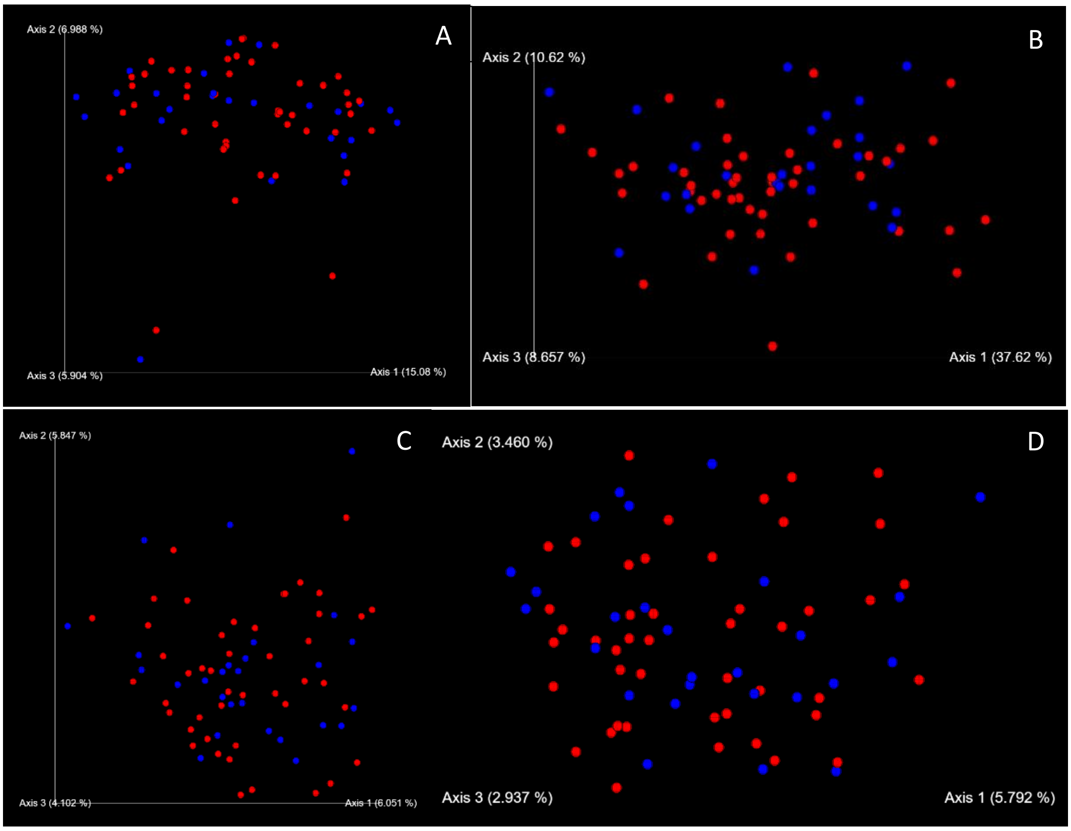

Supplement: Supplementary file 1 [file genes-13-00289-s001.zip › genes-1574098-supplementary.pdf]
